# Association of Symptoms of Posttraumatic Stress Disorder With Posttraumatic Psychological Growth Among US Veterans During the COVID-19 Pandemic

**DOI:** 10.1001/jamanetworkopen.2021.4972

**Published:** 2021-04-08

**Authors:** Robert H. Pietrzak, Jack Tsai, Steven M. Southwick

**Affiliations:** 1 US Department of Veterans Affairs National Center for Posttraumatic Stress Disorder, VA Connecticut Healthcare System, West Haven; 2 Department of Psychiatry, Yale School of Medicine, New Haven, Connecticut; 3 US Department of Veterans Affairs National Center on Homelessness Among Veterans, James A. Haley Veterans’ Hospital, Tampa, Florida; 4 School of Public Health, University of Texas Health Science Center at Houston, San Antonio Campus, San Antonio

## Abstract

This survey study uses self-reported data from the 2019-2020 National Health and Resilience in Veterans Study to assess the association of symptoms of posttraumatic stress disorder (PTSD) with posttraumatic psychological growth among US veterans during the COVID-19 pandemic.

## Introduction

Although extensive research has documented the negative psychiatric consequences of the COVID-19 pandemic,^1,2^ no study, to our knowledge, has examined whether the pandemic may be associated with positive psychological changes or posttraumatic growth (PTG). In addition to increasing risk for psychiatric illness, traumatic events may also stimulate PTG in the form of increased personal strength and appreciation of life, improved social relationships, spiritual changes, and new possibilities for one’s life.^3,4^ Posttraumatic growth is associated with better functioning^5^ and greater resilience to subsequent traumatic events^6^ in trauma survivors.

Military veterans may be at elevated risk for COVID-19–associated psychiatric issues given high rates of preexisting psychiatric conditions, such as posttraumatic stress disorder (PTSD) and suicidal ideation (SI). In this survey study, we analyzed data from a national sample of US military veterans to examine (1) the prevalence of COVID-19–associated PTG among veterans with and without COVID-19–associated PTSD symptoms and (2) the incremental association between PTG and SI during the pandemic.

## Methods

This survey study used data from a nationally representative sample of US veterans who participated in the 2019-2020 National Health and Resilience in Veterans Study. A total of 7860 veterans were invited to participate in the study, and 4069 (51.8%) completed a baseline survey (wave 1) between November 18, 2019, and March 8, 2020; of the latter group, 3078 veterans (75.6%) completed a 1-year follow-up survey between November 9 and December 19, 2020. All participants provided informed consent, and the study was approved by the Human Subjects Committee of the VA Connecticut Healthcare System. This study followed the American Association for Public Research (AAPOR) reporting guidelines.

The short form of the Posttraumatic Growth Inventory was used to assess COVID-19–associated PTG. Total scores and 5 subscales reflecting personal strength, relating to others, new possibilities, spiritual change, and appreciation of life^3^ were computed. A sample question regarding COVID-19–associated PTSD was “In the past month, how much were you bothered by: repeated, disturbing, and unwanted memories of the COVID-19 pandemic?” Descriptive statistics summarized the prevalence of self-reported PTG in the full sample and in veterans with and without a positive screen for COVID-19–associated PTSD. A multivariable logistic regression analysis was used to examine the association between PTG and SI, after adjustment for a broad range of background characteristics and pandemic-associated risk factors (Table). All frequencies are unweighted, and percentages are weighted to reflect the US veteran population. Data were analyzed using SPSS software version 27.0 (IBM Corp). All *P *values are 2-sided, and statistical significance was set at *P* < .05. Additional details regarding the study methods, assessment questions, and response options are provided in the eMethods in the Supplement.

**Table.** Sample Characteristics and Results of Multivariable Regression Model Examining Association Between Background and Pandemic-Associated Variables, COVID-19–Associated Posttraumatic Growth, and Current Suicidal Ideation Among US Military Veterans

Characteristic	Sample characteristics, No. (weighted %) (n = 3078)	Current suicidal ideation, OR (95% CI)^a^
Background characteristics		
Age, mean (SD), y	63.3 (14.7)	1.01 (0.99-1.02)
Sex		
Male	2730 (91.6)	1 [Reference]
Female	344 (8.4)	0.95 (0.54-1.69)
Race/ethnicity		
White, non-Hispanic	2541 (79.3)	1 [Reference]
Black, non-Hispanic	212 (10.3)	0.43 (0.19-0.95)^b^
Hispanic	216 (6.0)	0.99 (0.52-1.91)
Other, mixed race^c^	109 (4.4)	1.36 (0.62-3.00)
College graduate or higher education	1407 (34.2)	0.95 (0.64-1.41)
Married/partnered	2220 (74.0)	1.16 (0.78-1.75)
Retired	1733 (46.8)	1.14 (0.72-1.81)
Annual household income >$60 000	1851 (60.8)	0.78 (0.53-1.16)
Combat veteran	1051 (35.4)	1.24 (0.86-1.79)
Adverse childhood experiences	1.4 (1.9)	0.99 (0.91-1.08)
Sum of lifetime traumas^d^	8.9 (8.3)	0.97 (0.95-0.99)^b^
Traumas since wave 1 of survey^e^	1.0 (1.8)	1.07 (0.98-1.17)
Lifetime MDD or PTSD or both	590 (20.2)	0.88 (0.56-1.38)
Lifetime alcohol or drug use disorder or both	1265 (42.5)	1.29 (0.89-1.86)
Lifetime suicide attempt or suicidal ideation	350 (13.0)	9.76 (6.59-14.46)^f^
Baseline mental functioning score, weighted mean (SD)^g^	53.2 (7.9)	0.94 (0.92-0.96)^f^
Pandemic-associated risk factors		
COVID infection, self	233 (8.2)	1.95 (1.04-3.66)^b^
COVID infection, household member	198 (7.5)	1.00 (0.51-1.97)
COVID infection, nonhousehold member	1285 (41.4)	1.14 (0.79-1.65)
Daily COVID-19 media consumption, weighted mean (SD), h	1.6 (2.1)	1.02 (0.94-1.11)
COVID-19–associated worries, weighted mean (SD), factor score	0 (1.0)	1.20 (0.98-1.47)
COVID-19–associated social restriction stress, weighted mean (SD), factor score	0 (1.0)	1.15 (0.97-1.37)
COVID-19–associated financial difficulties, weighted mean (SD), factor score	0 (1.0)	1.46 (1.27-1.68)^f^
COVID-19–associated relationship difficulties, weighted mean (SD), factor score	0 (1.0)	1.12 (0.95-1.32)
Social engagement during pandemic, weighted mean (SD), factor score	0 (1.0)	0.94 (0.79-1.13)
COVID-19–associated PTSD symptoms	395 (12.8)	1.52 (0.92-2.49)
COVID-19–associated posttraumatic growth		
Appreciation of life	875 (29.3)	0.59 (0.35-0.98)^b^
Relating to others	691 (22.1)	0.58 (0.34-0.99)^b^
Personal strength	568 (19.3)	1.29 (0.75-2.24)
Spiritual change	543 (18.0)	1.11 (0.63-1.95)
New possibilities	401 (14.9)	0.64 (0.33-1.25)

Abbreviations: MDD, major depressive disorder; OR, odds ratio; PTSD, posttraumatic stress disorder.

^a^ A total of 236 veterans (weighted percentage, 8.0%) screened positive for suicidal ideation at the follow-up assessment.

^b^ Significant at 2-sided *P* < .05.

^c^ The other, mixed race category included Asian, American Indian and Alaska Native, Native Hawaiian and other Pacific Islander, and multiracial veterans.

^d^ Trauma history at wave 1 was evaluated using the Trauma History Screen, which assesses exposure to 14 potentially traumatic events occurring in childhood and adulthood.

^e^ Wave 1 of the survey was completed between November 21, 2019, and March 8, 2020.

^f^ Significant at 2-sided *P* < .001.

^g^ Scores range from 0 to 100, with 0 indicating the lowest or worst functioning and 100, the highest or best functioning.

## Results

Of the 3078 veterans who completed the 1-year follow-up survey, the mean (SD) age was 63.3 (14.7) years, 2730 (91.6%) were men, and 2541 (79.3%) were White, non-Hispanic individuals. A total of 395 veterans (12.8%) screened positive for COVID-19–associated PTSD symptoms. Of the 3078 respondents, 1328 veterans (weighted percentage, 43.3%) indicated they experienced moderate or greater levels of COVID-19–associated PTG, the most prevalent domains being appreciation of life, relating to others, and personal strength. Veterans who screened positive for COVID-19–associated PTSD symptoms were more likely than those who screened negative to endorse all aspects of PTG, with 276 veterans (weighted percentage, 71.9%) endorsing any COVID-19–associated PTG relative to 1052 veterans (weighted percentage, 39.1%) of those who screened negative for COVID-19–associated PTSD symptoms (Figure).

**Figure.**
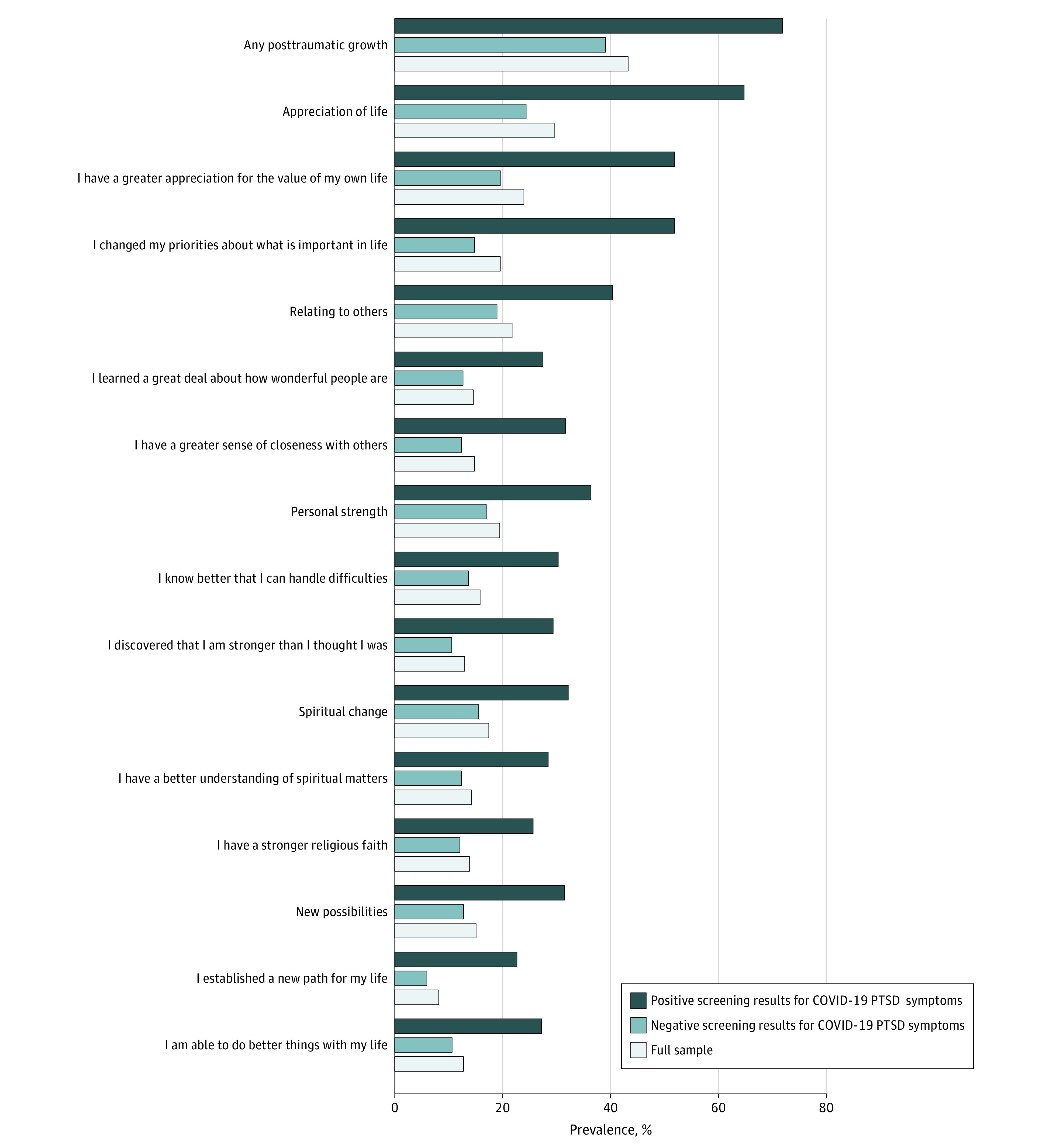
Prevalence of COVID-19–Associated Posttraumatic Growth Among 3078 US Military Veterans by COVID-19–Associated Posttraumatic Distress Disorder (PTSD) Screening Status A total of 395 veterans (12.8%) screened positive for symptoms of COVID-19–associated PTSD, and 2683 (87.2%) screened negative. The prevalence of self-reporting all aspects of posttraumatic growth was significantly higher in the group with positive screens. For all veterans, χ^2^_1_ values were greater than 45.29, and all *P* < .001.

A total of 219 veterans (7.7%) screened positive for SI at the baseline assessment and 236 (8.0%) at the follow-up assessment (McNemar test, 0.68; *P* = .41). After adjustment for background and pandemic-associated risk factors, COVID-19–associated improvements in appreciation of life (odds ratio, 0.59; 95% CI, 0.35-0.98) and relating to others (odds ratio, 0.58; 95% CI, 0.34-0.99) were independently associated with lower odds of SI at the follow-up assessment (Table).

## Discussion

Nearly 1 year into the pandemic, 43.3% of US military veterans reported moderate or greater levels of COVID-19–associated PTG, most commonly greater appreciation of life, improved social relationships, and increased personal strength. This prevalence is slightly lower than the 52.6% rate observed in a recent meta-analysis of PTG after a wide range of non–COVID-19–associated traumas,^4^ which may be partly attributable to our assessment of PTG in the midst of an ongoing pandemic.

Veterans who screened positive for COVID-19–associated PTSD symptoms had a markedly higher prevalence of PTG (weighted percentage, 71.9%), which is consistent with prior studies of non–COVID-19–associated traumas.^5^ Symptoms of PTSD associated with COVID-19 may prompt reflective processing of the pandemic, which may in turn help stimulate positive psychological changes.^3,5^ Greater COVID-19–associated improvements in appreciation of life and social relationships were associated with a significant reduction in the odds of SI. This finding aligns with those of prior work highlighting the clinical significance of PTG^5,6^ and underscores the importance of evaluating PTG-promoting interventions as part of suicide risk prevention and treatment efforts in veterans. Limitations of our study include the cross-sectional design and the use of self-reporting to assess study measures. Further research is needed to replicate these results and evaluate the efficacy of interventions to bolster PTG in promoting improved mental health in individuals affected by the ongoing pandemic.

## Notes

Supplement. **eMethods.** Supplemental Methods **eReferences.**
