# Supplementary material for: Association of Symptoms of Posttraumatic Stress Disorder With Posttraumatic Psychological Growth Among US Veterans During the COVID-19 Pandemic
Source: JAMA Netw Open. 2021 Apr 8;4(4):e214972. doi: 10.1001/jamanetworkopen.2021.4972 (PMC8033430; doi:10.1001/jamanetworkopen.2021.4972)
Supplement: Supplement. — eMethods. Supplemental Methods eReferences. [file jamanetwopen-e214972-s001.pdf]

Supplemental Online Content

Pietrzak RH, Tsai J, Southwick SM. Association of symptoms of posttraumatic stress disorder with posttraumatic psychological growth among US veterans during the COVID-19 pandemic. *JAMA Netw Open.* 2021;4(4):e214972. doi:10.1001/jamanetworkopen.2021.4972

eMethods. Supplemental Methods

eReferences.

This supplemental material has been provided by the authors to give readers additional information about their work.

© 2021 Pietrzak RH et al. *JAMA Network Open.*

eMethods. Supplemental Methods

Sample

The National Health and Resilience in Veterans Study (NHRVS) is a nationally representative survey of U.S. veterans A total of 4,069 veterans completed a Wave 1 / pre-pandemic (median completion date: 11/21/2019) survey prior to the first documented COVID-19 cases in the U.S. and 3,078 completed a Wave 2 peri-pandemic follow-up assessment one year later (median completion date: 11/14/2020). A total of 7,860 veterans were invited to participate in the study and 4,069 completed it (51.8% participation rate); of these 4,069 veterans, 3,929 (96.6%) remained in the survey panel when the follow-up survey was launched and 3,078 completed the follow-up survey (78.3% participation rate and 75.6% of the baseline cohort).

The NHRVS sample was drawn from KnowledgePanel, a research panel of more than 50,000 households that is maintained by Ipsos, a survey research firm. KnowledgePanel® is a probability-based, online non-volunteer access survey panel of a nationally representative sample of U.S. adults that covers approximately 98% of U.S. households. Panel members are recruited through national random samples, originally by telephone and now almost entirely by postal mail. Households are provided with access to the Internet and computer hardware if needed. KnowledgePanel® recruitment uses dual sampling frames that include both listed and unlisted telephone numbers, telephone and non-telephone households, and cell-phone-only households, as well as households with and without Internet access.

Demographic data of survey panel members are assessed regularly by Ipsos using the same set of questions used by the U.S. Census Bureau. Race/ethnicity was assessed via self-report using a standard set of questions used by the U.S. Census Bureau; this information was assessed in the current study to characterize the demographic composition of the sample and to adjust for any influence of race/ethnicity in a multivariable model examining the relation between aspects of posttraumatic growth and current suicidal ideation.

To permit generalizability of study results to the entire population of U.S. veterans, the Ipsos statistical team computed post-stratification weights using the following benchmark distributions of U.S. military veterans from the most recent (August 2019) Current Veteran Population Supplemental Survey of the U.S. Census Bureau's American Community Survey: age, gender, race/ethnicity, Census Region, metropolitan status, education, household income, branch of service, and years in service. An iterative proportional fitting (raking) procedure was used to produce the final post-stratification weights.

All participants provided informed consent and the study was approved by the Human Subjects Committee of the VA Connecticut Healthcare System.

© 2021 Pietrzak RH et al. *JAMA Network Open.*

American Association for Public Opinion Research Survey Disclosure Checklist:

Survey sponsor	U.S. Department of Veterans Affairs National Center for Posttraumatic Stress Disorder
Survey/Data collection supplier	Ipsos, Inc.
Population represented	U.S. military veterans in the United States
Sample size	3,078
Mode of data collection	Web-based survey panel
Type of sample (probability/non-probability)	Probability
Start and end dates of data collection	November 9, 2020 to December 19, 2020
Margin of sampling error for total sample	+/- 2.57 percentage points at the 95% confidence level
Are the data weighted?	Yes, using the following benchmark distributions of U.S. military veterans from the most recent (August 2019) Current Veteran Population Supplemental Survey of the U.S. Census Bureau's American Community Survey: gender, race/ethnicity, Census Region, metropolitan status, education, household income, branch of service, and years in service
Contact for more information	Robert H. Pietrzak, PhD, MPH<br><br>Research Psychologist, U.S. Department of Veterans Affairs National Center for PTSD<br><br>Associate Professor, Department of Psychiatry, Yale School of Medicine<br><br>(860) 638-7467,robert.pietrzak@yale.edu
Survey content may be obtained by contacting Dr. Pietrzak: robert.pietrzak@yale.edu	

Assessments

COVID-19-associated posttraumatic growth. The Posttraumatic Growth Inventory-Short Form[1] was used to assess COVID-19-associated PTG at Wave 2 (α=0.92). Total scores and five subscales reflecting personal strength, relating to others, new possibilities, spiritual change, and appreciation of life were computed. Sample item: "Please indicate the degree to which you experienced these changes in your life as a result of the Coronavirus/COVID-19 pandemic: I have a greater sense of closeness with others." Endorsements of "moderate," "great," or "very great" growth were indicative of PTG.

© 2021 Pietrzak RH et al. *JAMA Network Open.*

Please indicate for each of the statements below the degree to which you experienced these changes in your life as a result of the Coronavirus/COVID-19 pandemic:

Items

1. I changed my priorities about what is important in life.
2. I have a greater appreciation for the value of my own life.
3. I am able to do better things with my life.
4. I have a better understanding of spiritual matters.
5. I have a greater sense of closeness with others.
6. I established a new path for my life.
7. I know better that I can handle difficulties.
8. I have a stronger religious faith.
9. I discovered that I'm stronger than I thought I was.
10. I learned a great deal about how wonderful people are.

Response Options

I DID NOT experience this change as a result of the Coronavirus/COVID-19 pandemic.
I experienced this change to a VERY SMALL degree as a result of the Coronavirus/COVID-19 pandemic.
I experienced this change to a SMALL degree as a result of the Coronavirus/COVID-19 pandemic.
I experienced this change to a MODERATE degree as a result of the Coronavirus/COVID-19 pandemic.
I experienced this change to a GREAT degree as a result of the Coronavirus/COVID-19 pandemic.
I experienced this change to a VERY GREAT degree as a result of the Coronavirus/COVID-19 pandemic

Trauma history at Wave 1 was assessed using the Trauma History Screen,[2] which assesses exposure to 14 potentially traumatic events occurring in childhood and adulthood. Adverse childhood experiences at Wave 1 were assessed using the Adverse Childhood Experiences Scale.[3] Past-year trauma history at Wave 2 was assessed at the follow-up assessment using the Life Events Checklist for DSM-5.[4]

Wave 1 lifetime major depressive disorder, alcohol and drug disorder were assessed using adapted self-report modules of the Mini Neuropsychiatric Interview.[5]

Mental functioning at Wave 1 was assessed using the Short Form 8 Health Survey,[6] which assesses vitality (energy and fatigue), general mental health and well-being, limitations in usual activities because of emotional problems, and limitations in social activities because of emotional or physical problems.

Wave 1 PTSD symptoms. The PTSD Checklist-Specific Stressor Version[7] was used to assess lifetime history of PTSD associated to veterans' worst traumatic event endorsed on the Trauma History Screen.

© 2021 Pietrzak RH et al. *JAMA Network Open.*

COVID-related PTSD symptoms. A brief, 4-item version of the PTSD Checklist for DSM-5[8] was used to assess COVID-19-associated PTSD symptoms at Wave 2 (score range=0-16; α=0.76; sample item: "Thinking about the Coronavirus/COVID-19 pandemic, please indicate how much you have been bothered by repeated, disturbing, and unwanted memories of the COVID-19 pandemic"); a score ≥ 5 is indicative of a positive screen for PTSD.

COVID-19-associated exposures and worries. At Wave 2, COVID-19 infection status of veterans, household members, and non-household members were assessed using a questionnaire developed by the National Center for PTSD.[9] COVID-19 infection status was assessed using the question: "Have any of the following people ever had COVID-19? You ; Someone who lives in your home ; and Someone close to you who does not live in your home (for example, a friend, a family member who does not live with you, a co-worker, or child care provider." Response options were: Yes, tested positive for COVID-19 or COVID-19 antibodies; Probably, but never received a diagnosis; and No, have never had COVID-19 or a suspected case.

Questions from the Coronavirus Health Impact Survey[10] were used to assess COVID-19-associated worries and concerns at Wave 2. Factor analysis revealed that these items loaded on five factors (eigenvalues=1.01-4.94): COVID-19-associated worries (e.g., "In the past month, how worried have you been about being infected with coronavirus?"); COVID-19 social restriction stress (e.g., "How stressful have these changes in social contacts been for you?"); COVID-19-associated financial hardship (e.g., "In the past month, to what degree have changes associated to the pandemic created financial problems for you or your family?"); COVID-19-associated relationship difficulties (e.g., "Has the quality of the relationships between you and members of your family changed?"); and COVID-19-associated social engagement (e.g., "In the past month, how many people, from outside of your household, have you had an in-person conversation with?").

Current suicidal ideation was assessed at Waves 1 and 2 using two items adapted from item 9 of the Patient Health Questionnaire-9,[11] which asked participants to report suicidal ideation during the prior two weeks. A positive screen for current suicidal ideation was indicated by a response of "several days," "more than half the days," or "nearly every day" to at least one of the following questions: "How often have you been bothered by thoughts that you might be better off dead?" and "How often have you been bothered by thoughts of hurting yourself in some way?"

© 2021 Pietrzak RH et al. *JAMA Network Open.*

eReferences.

1. Cann A, Calhoun LG, Tedeschi RG, et al. A short form of the Posttraumatic Growth Inventory. *Anxiety Stress Coping*. 2010;23(2):127-137.
2. Carlson EB, Smith SR, Palmieri PA, et al. Development and validation of a brief self-report measure of trauma exposure: the Trauma History Screen. *Psychol Assess*. 23(2):463-477.
3. Felitti VJ, Anda RF, Nordenberg D, et al. Relationship of childhood abuse and household dysfunction to many of the leading causes of death in adults. The Adverse Childhood Experiences (ACE) Study. *Am J Prev Med.* 1998;14(4):245-258.
4. Weathers FW, Blake DD, Schnurr PP, et al.: The Life Events Checklist for DSM-5 (LEC-5). 2019. Instrument available from the National Center for PTSD at www.ptsd.va.gov.
5. Sheehan DV, Lecrubier Y, Sheehan KH, et al. The Mini-International Neuropsychiatric Interview (MINI): the development and validation of a structured diagnostic psychiatric interview for DSM-IV and ICD-10. *J Clin Psychiatry*. 1998; 59 Suppl 20: 22-33.
6. Ware JE, Kosinski M, Dewey JE, et al: How to Score and Interpret Single-Item Health Status Measures: A Manual for Users of the SF-8 Health Survey. Lincoln, RI, QualityMetric, Inc., 2001.
7. Weathers F, Litz B, Herman D, Huska J, Keane T. The PTSD Checklist (PCL): Reliability, Validity, and Diagnostic Utility. Paper presented at the Annual Convention of the International Society for Traumatic Stress Studies, San Antonio, TX. October 1993.
8. Geier TJ, Hunt JC, Hanson JL, et al. Validation of abbreviated four- and eight-item versions of the PTSD Checklist for DSM-5 in a traumatically injured sample. *J Trauma Stress*. 2020;33(3):218-226.
9. National Center for PTSD. *Assessment of Exposure to COVID-19.* Internal working document. 2020. Available from the National Center for PTSD at www.ptsd.va.gov.
10. National Institute of Mental Health Intramural Research Program Mood Spectrum Collaboration, Child Mind Institute of the NYS Nathan S. Kline Institute for Psychiatric Research. The CoRonavIruS Health Impact Survey (CRISIS). 2020; https://www.nlm.nih.gov/dr2/CRISIS_Adult_Self-Report_Baseline_Current_Form_V0.3.pdf.
11. Kroenke K, Spitzer RL, Williams JBW. The PHQ-9: validity of a brief depression severity measure. *J Gen Intern Med*. 2001;16(9):606-613.

© 2021 Pietrzak RH et al. *JAMA Network Open.*
